# Effects of acromegaly treatment on left ventricular systolic function assessed by speckle tracking echocardiography in relation to sex differences: results from a prospective single center study

**DOI:** 10.3389/fendo.2023.1154615

**Published:** 2023-05-08

**Authors:** Agata Popielarz-Grygalewicz, Maria Stelmachowska-Banaś, Dorota Raczkiewicz, Izabella Czajka-Oraniec, Grzegorz Zieliński, Wacław Kochman, Marek Dąbrowski, Wojciech Zgliczyński

**Affiliations:** ^1^ Department of Cardiology, The Centre of Postgraduate Medical Education, Warsaw, Poland; ^2^ Department of Endocrinology, The Centre of Postgraduate Medical Education, Warsaw, Poland; ^3^ Department of Medical Statistics, School of Public Health, The Centre of Postgraduate Medical Education, Warsaw, Poland; ^4^ Department of Neurosurgery, Military Institute of Medicine, Warsaw, Poland; ^5^ Department of Cardiology, Bielanski Hospital, Warsaw, Poland

**Keywords:** acromegaly, global longitudinal strain (GLS), speckle tracking echocardiography (STE), somatostatin receptor ligands (SRL), transsphenoidal pituitary surgery

## Abstract

**Background:**

Despite the preserved LVEF, patients with acromegaly are characterized by subclinical systolic dysfunction i.e., abnormal global longitudinal strain (GLS) assessed by speckle tracking echocardiography (STE). The effect of acromegaly treatment on LV systolic function assessed by STE, has not been evaluated so far.

**Patients and methods:**

Thirty-two naïve acromegalic patients without detectable heart disease were enrolled in a prospective, single-center study. 2D-Echocardiography and STE were performed at diagnosis, 3&6 months on preoperative somatostatin receptor ligand (SRL) treatment and 3 months after transsphenoidal surgery (TSS).

**Results:**

Treatment with SRL resulted in reduction in median (IQR) GH&IGF-1 levels after 3 months, from 9.1(3.2-21.9) to 1.8(0.9-5.2) ng/mL (p<0.001) and from 3.2(2.3-4.3) to 1.5(1.1-2.5) xULN (p<0.001), respectively. Biochemical control on SRL was achieved in 25.8% of patients after 6 months and complete surgical remission was achieved in 41.7% of patients. TSS resulted in decrease in median (IQR) IGF-1 compared to IGF-1 levels on SRL treatment: from 1.5(1.2-2.5) to 1.3(1.0-1.6) xULN (p=0.003). Females had lower IGF-1 levels at baseline, on SRL and after TSS compared to males. The median end diastolic and end systolic left ventricle volumes were normal. Almost half of the patients (46.9%) had increased LVMi, however the median value of LVMi was normal in both sex groups: 99g/m^2^ in males and 94g/m^2^ in females. Most patients (78.1%) had increased LAVi and the median value was 41.8mL/m^2^. At baseline 50% of patients, mostly men (62.5% vs. 37.5%) had GLS values higher than -20%. There was a positive correlation between baseline GLS and BMI r=0.446 (p=0.011) and BSA r=0.411 (p=0.019). The median GLS significantly improved after 3 months of SRL treatment compared to baseline: -20.4% vs. -20.0% (p=0.045). The median GLS was lower in patients with surgical remission compared to patients with elevated GH&IGF-1 levels: -22.5% vs. -19.8% (p=0.029). There was a positive correlation between GLS and IGF-1 levels after TSS r=0.570 (p=0.007).

**Conclusion:**

The greatest beneficial effect of acromegaly treatment on LV systolic function is visible already after 3 months of preoperative SRL treatment, especially in women. Patients with surgical remission have better GLS compared to patients with persistent acromegaly.

## Introduction

Acromegaly is a systemic disease that affects multiple organs including the cardiovascular system (1). Recent data show that mortality rate among patients with well-controlled acromegaly is similar to that of the normal aging population (1) and there is a shift from cardiovascular disease to cancer as the leading cause of mortality (2, 3). Although cardiovascular morbidity has improved significantly in recent years, cardiovascular disease is still an important cause of mortality among patients with acromegaly. There are studies showing an increased cardiovascular mortality among acromegaly patients compared to the general population, particularly in females (2, 4). It remains unclear how much those changes in morbidity and mortality are influenced by improved methods of treatment of acromegaly and its comorbidities or overall improved cardiac care and more strict cardiovascular risk management (5).

Acromegaly can cause a typical cardiomyopathy characterized by mainly left ventricular hypertrophy (LVH) that is associated with mildly impaired diastolic function, which can progress to systolic dysfunction (6). However, most echocardiographic studies have shown that systolic function in acromegaly measured by ejection fraction (EF) is preserved, and that symptomatic heart failure is an uncommon and late complication (5, 7, 8). Therefore, identifying patients with acromegaly who have increased cardiovascular risk is the main challenge in avoiding this late phase of acromegalic cardiomyopathy. Two-dimensional speckle-tracking echocardiography (2D-STE) is a modern technique that allows the evaluation of longitudinal, radial, and circumferential deformation and offers a more sensitive assessment of myocardial contractility, especially in patients with preserved EF (9). The global longitudinal strain (GLS) is a well validated, reproducible tool for the measurement of global left ventricular (LV) systolic function and provides relevant evidence on the diagnostic and prognostic implications (10, 11). Reduction in GLS predicts worse cardiovascular outcomes (12, 13) and evaluation of such reduction has been applied to the early detection of heart valve diseases, myocardial ischemia, hypertrophic cardiomyopathy, and cardiotoxicity during cancer therapy (14) as well as in the preclinical detection of cardiac involvement in the systemic, metabolic and endocrine diseases (13, 15–17). To date, there have been only a few studies evaluating LV function using STE in patients with active acromegaly and preserved EF (8, 18–20). However, the results have been inconsistent and only some studies showed LV subclinical systolic dysfunction (18, 21), while in other studies GLS did not differ between patients with acromegaly and the control groups (19, 20). The discrepancies in these results may be due to inhomogeneous groups of patients with acromegaly and previous treatment of acromegaly in these patients. A beneficial effect of acromegaly treatment on subclinical LV dysfunction cannot be ruled out, even in patients who fail to achieve full biochemical control of the disease. However, the effect of acromegaly treatment on LV systolic function assessed by STE, has not been evaluated so far. Thus the aim of our study was to investigate the effect of acromegaly treatment on LV function measured by STE in naïve patients, considering potential sex differences.

## Patients and methods

### Study design and patients

This was a prospective, single-center study. The study group consisted of 35 consecutive newly diagnosed acromegaly patients admitted to the Department of Endocrinology at the Center of Postgraduate Medical Education in Warsaw, Poland, from January 2018 to July 2020. Only adult patients (age ≥ 18 years) were enrolled to this study. Acromegaly was diagnosed based on typical clinical features associated with elevated serum IGF-1 levels for age and sex and lack of GH suppression below 0.4 ng/mL during a 75 g oral glucose tolerance test (OGTT), and positive pituitary magnetic resonance (MRI) findings.

After the diagnosis of acromegaly, each patient received a preoperative treatment with the first-generation somatostatin receptor ligand (SRL) (lanreotide autogel 120 mg every 4 weeks s.c.) while waiting for transsphenoidal pituitary surgery (TSS). Biochemical response to the medical therapy was assessed after a 6-month treatment period for 31 patients. Good response to first-generation SRLs was defined as fasting GH ≤1 ng/mL and IGF-1 ≤ the upper limit of normal (ULN) for age and sex on treatment with a maximum dose of SRL.

Surgical remission was defined as IGF-1 concentration below the ULN for sex and age-matched groups and suppression of GH on OGTT below 0.4 ng/mL assessed 3 months after TSS. Out of 32 enrolled patients 24 underwent the TSS (75%) during the study after preoperative treatment with SRL.

All patients were operated on by one neurosurgeon experienced in pituitary surgeries from the Department of Neurosurgery, Military Institute of Medicine in Warsaw, Poland.

Each patient underwent echocardiography at the time of diagnosis of acromegaly, 3 and 6 months after the initiation of treatment with SRL and 3 months after TSS. Patients with a known coronary artery disease, with a history of myocardial infarction or myocarditis, with impairment of LV systolic function (EF < 54% in women and < 52% in men), and moderate to severe valvular disease were not included in this study.

From the initial number of 35 patients with newly diagnosed acromegaly enrolled to this study, 32 patients were included to the final analysis. Three patients were excluded: 1 with a history of myocardial infarction, 1 with multivessel coronary artery disease and concomitant ventricular tachycardias and 1 with heart failure and reduced EF. Additionally, 28 out of 32 enrolled patients underwent carotid Doppler ultrasonography at the diagnosis of acromegaly.

The project received the approval of the Bioethics Committee of the Center of Postgraduate Medical Education in Warsaw, Poland.

### Clinical and biochemical parameters

The presumed duration of acromegaly was assessed by comparing old photographs and conducting interviews. Body mass index (BMI) was calculated using the formula weight (kg) divided by height squared (m^2^), body surface area (BSA) was calculated according to the DuBois formula, and systolic and diastolic blood pressures were measured in millimeters of mercury (mmHg). Arterial hypertension was defined as a systolic blood pressure of ≥ 140 mmHg and/or diastolic blood pressure of ≥ 90 mmHg or the treatment of previously diagnosed hypertension. Plasma glucose, glycosylated hemoglobin A1c, total cholesterol, high-density lipoprotein cholesterol, and triglycerides were measured in the morning after a 12-hour fasting period. Low density lipoprotein cholesterol was calculated by the Friedewald formula. Diabetes or prediabetes were diagnosed according to the recent criteria (22). Gonadal function in females was assessed on the basis of menstrual status and FSH/LH and estradiol levels.

Blood samples taken for GH and IGF-1 were analyzed with chemiluminescence immunoassay using the LIAISON^®^ XL analyzer (DiaSorin, Italy). The GH assay has a sensitivity of 0.05 ng/mL, an intra-assay coefficient of variation (CV) of 1.93% for a GH concentration of 1.18 ng/mL and inter-assay CV of. 3.77% for a GH concentration of 1.11 ng/mL. The intra-assay CV is 4.59% for an IGF-1 concentration of 189.3 ng/mL and inter-assay CV is 4.3% for an IGF-1 concentration of 202.6 ng/mL.

### Standard echocardiography

Each patient with confirmed acromegaly was referred to the Echocardiography Laboratory of the Department of Cardiology at the Center of Postgraduate Medical Education, Warsaw.

Standard two-dimensional transthoracic echocardiographic study and Speckle Tracking Echocardiography were performed at four time points as detailed above.

All studies were performed by the same experienced investigator. Echocardiography exam was done using Vivid 9 device (Horten, Norway). A sector transducer with a frequency of 3.2 MHz was used. The images were stored digitally for later offline analysis using dedicated software (EchoPac PC, workstation version 113, GE Medical Systems). LV and left atrial (LA) volumes were calculated using the apical four- and two-chamber views. LV volumes were normalized by body surface area (BSA) and the value of left ventricular end diastolic volume (LVEDVi) ≤ 61mL/m^2^ for women and ≤ 74mL/m^2^ for men, were accepted as normal. The cut-off points for indexed left ventricular end-systolic volume (LVESVi) were as follows: ≤24 mL/m^2^ for women, ≤ 31mL/m^2^ for men.

Left atrium volume (LAV) was also indexed to BSA, and the value of LAVi ≤ 34mL/m^2^ was considered normal for both men and women.

LV EF was calculated using the biplane Simpson formula and value ≥ 52% for men, and ≥ 54% for women were considered normal. The LV mass (LVM) was calculated using the linear method and was indexed in accordance with BSA to obtain the left ventricular mass index (LVMi) value. This parameter was calculated by using Penncube method according to Devereux’s formula: LVMi (g/m2) = (1.04 × (IVST + LVID + PWT)3 − (LVID)3 − 13.6)/BSA (Devereux et al., 1986). LVMi ≤ 95 g/m2 in women and ≤ 115 g/m2 in men were accepted as cut-off points for normal.

These echocardiographic parameters were measured in accordance with Recommendations for Cardiac Chamber Quantification by Echocardiography in Adults: An Update from the American Society of Echocardiography and the European Association of Cardiovascular Imaging (23).

LV diastolic function was measured using pulse Doppler from the apical 4-chamber view (assessing mitral inflow velocities as E wave and A wave) and using tissue Doppler imaging (assessing mitral annulus septal and lateral velocities as E’med and E’lat). To calculate the ratio E/e’ the average value of the septal and lateral mitral annulus velocities was used.

E’lat was not measured in 5 patients, and in this group E’ med was used to calculate the E/E’ ratio. E’med value above and equal to 7 and E’lat above and equal to 10 were considered normal.

E/E’ ratio of less than 10 was considered the cut-off for normal. The value of the E/A ratio in the range of 0.8 to 2 is considered normal, and values below 0.8 and above 2 were interpreted in the context of other parameters, including age.

These pulse and tissue Doppler parameters were evaluated and interpreted in accordance with Recommendations for the Evaluation of Left Ventricular Diastolic Function by Echocardiography: An Update from the American Society of Echocardiography and the European Association of Cardiovascular Imaging (24).

### Speckle tracking echocardiography

The images for GLS measurement were stored for later offline analysis using dedicated software (EchoPac PC, workstation version 113, GE Medical Systems). We used the automated function imaging (AFI) application to evaluate GLS.

The analysis involved ECG-gated digital images in four-, three-, and two-chamber apical views, and a high temporal resolution of 50–60 frames per second was obtained to assure acoustic-marker tracking. The LV walls were divided into six segments in each apical view, and strain values were assessed for each LV segment. Only the images with appropriate tracking in all the myocardial segments were used in the analysis. GLS was the average of the values that were obtained for three apical views. GLS is a negative percentage number and indicates fiber shortening. In accordance with the guidelines, a value of GLS of –20% or lower (i.e. more negative) was taken as normal in our study (23).

### Carotid doppler ultrasonography

Carotid Doppler ultrasound was performed using Vivid 9 device (Horten, Norway). A linear transducer with a frequency of 9.5 MHz was used (GE 11L). The measurements of the carotid intima media thickness (CIMT) were obtained 5-10 mm from the bifurcation around the posterior wall. To optimize the image zoom was used. Color Doppler and pulse Doppler ultrasonography have also been used. CIMT values ≤0.9 mm were considered normal, and CIMT values ≥ 1.5mm were considered as atherosclerosis plaque. Intermediate values (1.0-1.4mm) in our study were classified as abnormal. This interpretation is in accordance with:

1. Recommendations for the Assessment of Carotid Arterial Plaque by Ultrasound for the Characterization of Atherosclerosis and Evaluation of Cardiovascular Risk: From the American Society of Echocardiography (25).

2. Standards of ultrasound examinations of the Polish Ultrasound Society - update. Examination of the intracranial sections of the carotid and vertebral arteries (26).

### Statistical methods

The data were statistically analyzed using STATISTICA 13 software.

Median and interquartile range (IQR) (25% - 75%) were estimated for numerical variables, while counts (n) and percentages (%) of the occurrence of items for categorical variables.

Mann-Whitney U test was used to compare numerical variables between two categories of categorical characteristics. Kruskal-Wallis H test was used to compare numerical variables between three categories of categorical characteristics. Fisher exact test was used to compare categorical variables between categories of categorical characteristics. Wilcoxon signed ranks test was used to compare numerical variables between baseline and after 3 and 6 months of SRL treatment, and 3 months after TSS. Scatter plots and Pearson’s correlation coefficient were used to correlate numerical variables between each other. The missing data were omitted in all the analyses. The significance level was assumed at 0.05.

## Results

### Clinical and biochemical characteristics of the study group

The median age of 32 enrolled patients with newly diagnosed acromegaly was 52.5 (IQR 42-60) years and there were 16 (50%) males. The median duration of symptoms until the diagnosis of acromegaly was 10 (IQR 5-10) years. Seventeen patients (53.13%) had hypertension at acromegaly diagnosis and the majority (82.3%) of them were treated either with angiotensin converting enzyme inhibitors (ACEI) or angiotensin receptor blockers (ARB). Only 18.75% of the patients had normal glucose tolerance, 21.88% had diabetes and 59.38% - prediabetes. Dyslipidemia was present in 37.5% of acromegalic patients and 25% had carotid artery atherosclerosis diagnosed by Doppler ultrasonography. None of the patients with confirmed atherosclerosis had significant stenoses above 50% of the vessel lumen. Seven females (46.7%) were considered to be eugonadal because they had regular menstrual cycles and/or conceived spontaneously and had normal FSH/LH and estradiol concentrations at diagnosis while 9 females (56.3%) were considered hypogonadal either due to menopause or due to gonadotroph deficiency.

There were no statistically significant differences in the occurrence of comorbidities and the duration of symptoms to diagnosis between women and men. Men had statistically significant higher BMI and BSA compared to women: 29.0 (IQR 27.4-30.5) vs. 26.0 (21.8-27.7) kg/m^2^, p=0.006 and 2.2 (2.1-2.3) vs. 1.8 (1.7-1.8) m^2^, p<0.001.

The median baseline GH concentration was 9.1 (IQR 3.2-21.9) ng/mL, nadir GH on OGTT was 4.9 (IQR 1.9-21.1) ng/mL and IGF-1 concentration was 3.2 (IQR 2.3-4.3) x ULN for sex and age. Men had significantly higher biochemical activity of acromegaly compared to women measured by IGF-1 concentration: 3.9 (IQR 3.0-4.7) x ULN vs. 2.8 (IQR 2.1-3.3) x ULN, p=0.027. The detailed baseline clinical and biochemical characteristics of the studied group are presented in **Table 1**.

**Table 1:** Clinical and hormonal characteristics of acromegalic patients (N=32), at baseline.

Variable	Total (N=32)	Men (N=16)	Women (N=16)	p
Age at diagnosis (years), median (IQR)	53 (42–60)	53 (41-58)	51 (42-61)	0.748
BMI (kg/m^2^), median (IQR)	27.9 (25.8-29.8)	29.0 (27.4-30.5)	26.0 (21.8-27.7)	**0.006**
Disease duration (years), median (IQR)	10 (5-10)	10 (5-13)	5 (3-10)	0.117
BSA (m^2^), median (IQR)	2.0 (1.8-2.2)	2.2 (2.1-2.3)	1.8 (1.7-1.8)	**<0.001**
Hypertension, n (%), yes	17 (53.13)	10 (62.50)	7 (43.75)	0.479
Diabetes status, n (%), diabetes	7 (21.88)	2 (12.50)	5 (31.25)	0.492
prediabetes	19 (59.38)	11 (68.75)	8 (50.00)	
no diabetes	6 (18.75)	3 (18.75)	3 (18.75)	
Dyslipidemia, n (%), yes	12 (37.50)	7 (43.75)	5 (31.25)	0.716
Carotid arteries, n (%), atherosclerosis *	7 (25.00)	3 (23.08)	4 (26.67)	0.999
normal	18 (64.29)	9 (69.23)	9 (60.00)	
abnormal	3 (10.71)	1 (7.69)	2 (13.33)	
Fasting GH (ng/mL), median (IQR)	9.1 (3.2-21.9)	11.6 (3.3-27.1)	6.4 (2.5-20.8)	0.665
Nadir GH during 75g OGTT (ng/mL), median (IQR)	4.9 (1.9-21.1)	6.8 (2.4-32.1)	4.7 (1.8-13.4)	0.621
IGF-1 (ng/mL), median (IQR)	755 (558-942)	803 (643-1051)	732 (508-842)	0.181
IGF-1 (x ULN), median (IQR)	3.2 (2.3-4.3)	3.9 (3.0-4.7)	2.8 (2.1-3.3)	**0.027**

*no data for 4 patients (3 men and 1 woman), IQR, interquartile range; BMI, body mass index; BSA, body surface area; ULN, upper limit of normal; p for U – Mann-Whitney test to compare numerical variables between genders, or for Fisher exact test to compare categorical variables between genders. The bold values are statistically significant.

### Hormonal parameters during acromegaly treatment

**Figure 1:**
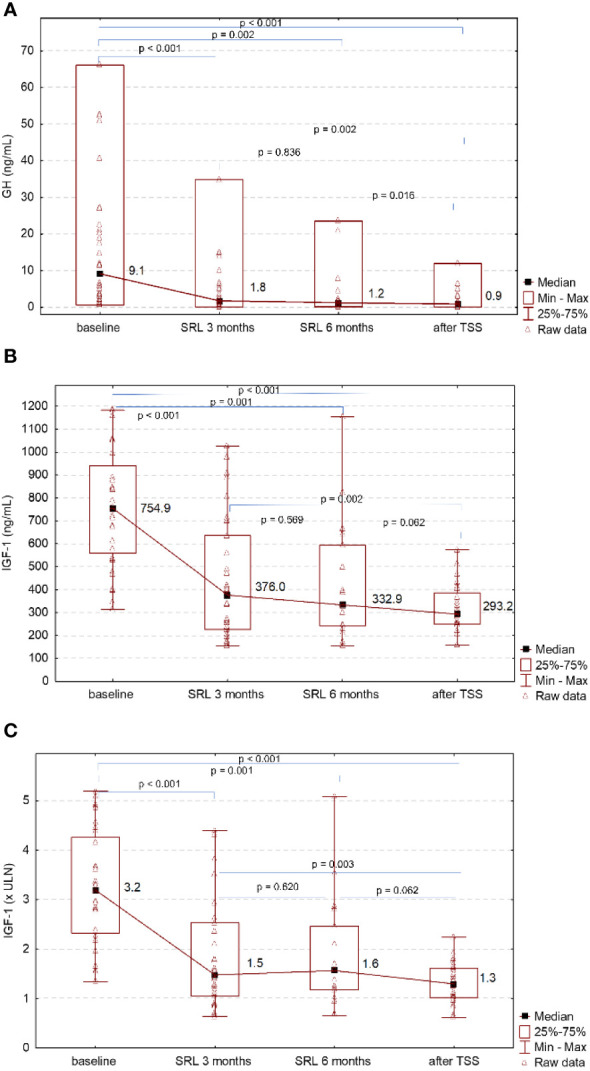
**(A)** Changes of GH at baseline, after 3 and 6 months of SRL treatment and 3 months after TSS. **(B)** Changes of IGF-1 at baseline, after 3 and 6 months of SRL treatment and 3 months after TSS. **(C)** Changes of IGF-1xULN at baseline, after 3 and 6 months of SRL treatment and 3 months after TSS.

After preoperative SRL treatment the median fasting GH concentration decreased significantly after 3 months since the initiation of SRL from 9.1 (IQR 3.2-21.9) ng/mL to 1.8 (IQR 0.9-5.2) ng/mL, p<0.001. Longer treatment with SRL did not result in further significant decrease in GH concentration (p=0.836). However, a significant further decrease in GH concentration was reached after TSS: from 1.2 (IQR 0.8-2.1) ng/mL after 6 months SRL treatment to 0.9 (IQR 0.3-2.4) ng/mL after TSS, p=0.016 (**Figure 1A**). The median GH nadir on OGTT decreased from 4.9 (IQR 1.9-21.1) ng/mL at baseline to 0.5 (IQR 0.2-1.2) ng/mL after TSS, p<0.001.

The baseline median IGF-1 concentration was 3.2 (IQR 2.3-4.3) x ULN and significantly decreased 3 months after SRL initiation to 1.5 (IQR 1.1-2.5) x ULN, p<0.001. Longer treatment with SRL did not lead to further decrease in IGF-1 concentration (p=0.620). Biochemical control after 6 months of SRL treatment was achieved in 8 out of 31 patients (25.81%). A significant decrease in IGF-1 levels compared to IGF-1 levels on SRL treatment was observed 3 months after TSS: from 1.5 (IQR 1.2-2.5) x ULN after 3 months SRL treatment to 1.3 (IQR 1.0-1.6) x ULN after TSS, p=0.003 (**Figures 1B, C**).

**Figure 2:**
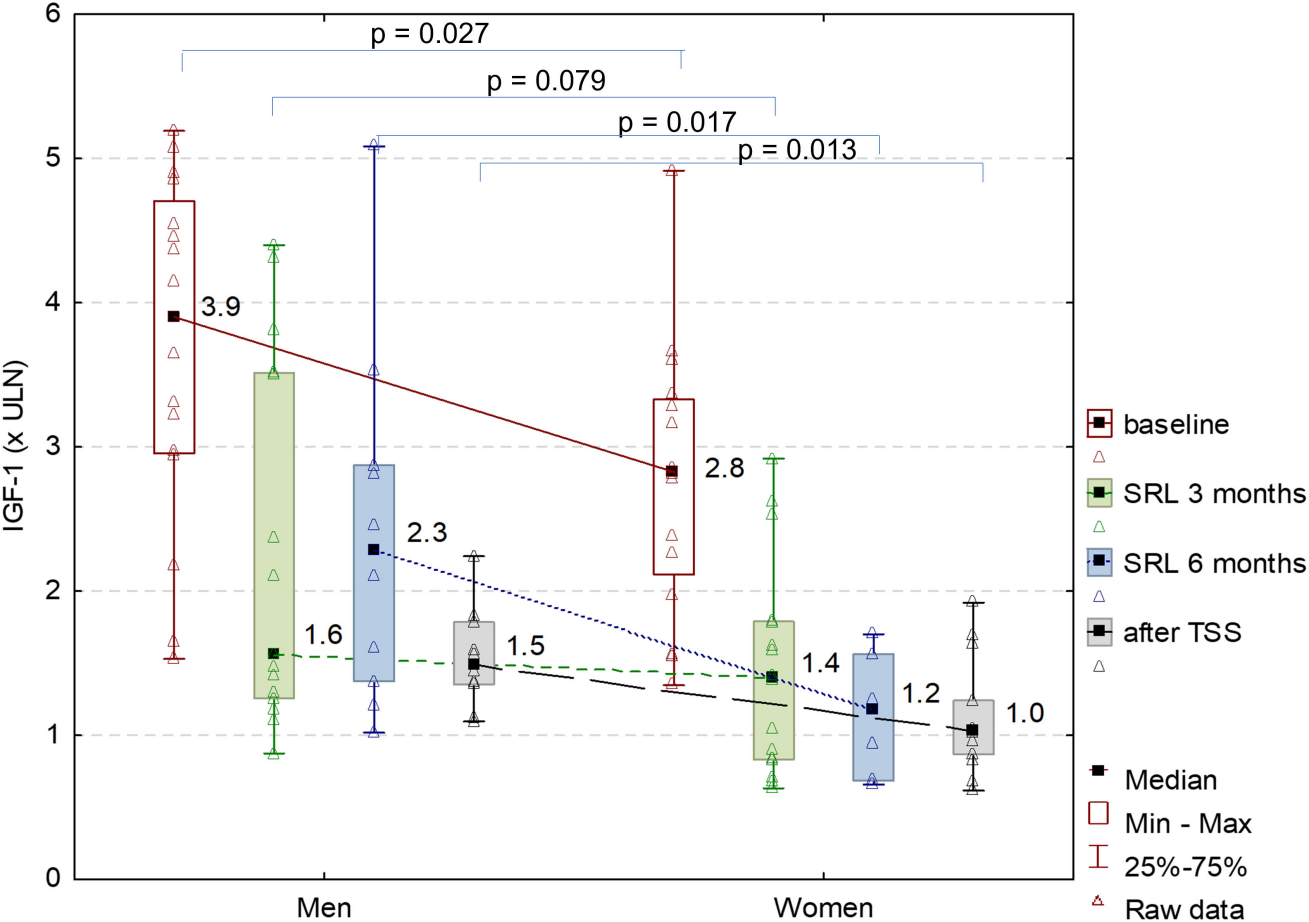
IGF-1xULN at baseline, after 3 and 6 months of SRL treatment and 3 months after TSS, by gender.

Complete surgical remission after TSS was achieved in 10 out of 24 operated patients (41.67%). Females had significantly lower IGF-1 levels not only before treatment but also after 6 months of SRL and 3 months after TSS compared to males (**Figure 2**).

The detailed characteristics of biochemical parameters during treatment for each patient is presented in **Supplementary Figures 1A–C**.

### Standard echocardiographic evaluation

The majority of patients had normal end diastolic and end systolic LV volumes. Only 9.38% of patients had increased end diastolic volume and 12.5% had increased end systolic volume. The median values of these parameters were within normal limits: LVEDVi 56.4mL/m^2^ in males and 44.5mL/m^2^ in females, LVESVi 23.4mL/m^2^ in males, and 16.7mL/m^2^ in females.

46.88% of patients (8 females and 7 males) had abnormal LVMi, however the median values of LVMi remained normal in both sex groups: 99g/m^2^ in males and 94g/m^2^ in females.

Most patients (78.13%; 14 females and 11 males) had abnormal LAVi and the median was 41.8mL/m^2^. Interestingly, in the group of women this value was higher compared to men (43mL/m^2^ vs. 38.5mL/m^2^), but the difference was not statistically significant (p=0.3). More than 50% of the patients had abnormal diastolic function assessed by TDI; the median E’med was 6 cm/s and E’lat was 9 cm/s, these values were mildly lower than normal, and the median E/E’ ratio was 10 and it remained within the ULN. Most patients (68.75%) had normal mitral inflow as assessed by pulse Doppler; the median E/A ratio was 0.9 and it was normal. All patients included in the study had normal EF, the median EF was 64% (62% in males and 66% in females). The baseline echocardiography parameters are presented in **Table 2**.

**Table 2:** Echocardiography parameters in acromegalic patients (N=32), at baseline.

Variable	Total (N=32)	Men (N=16)	Women (N=16)	p
Cardiac chamber size				
LVEDV (mL), median (IQR)	105 (81-124)	119 (99-141)	83 (72-109)	**0.001**
above normal, n (%)	8 (25.00)	3 (18.75)	5 (31.25)	0.685
LVEDV/BSA (mL/m^2^), median (IQR)	50.7 (43.2-63.4)	56.4 (47.3-66.7)	44.5 (42.4-57.4)	0.118
above normal, n (%)	3 (9.38)	0 (0.00)	3 (18.75)	0.226
LVESV (mL), median (IQR)	39 (29-53)	50 (38-56)	30 (26-41)	**0.003**
above normal, n (%)	6 (18.75)	3 (18.75)	3 (18.75)	0.999
LVESV/BSA (mL/m^2^), median (IQR)	20.0 (15.8-24.1)	23.4 (18.2-24.4)	16.7 (14.8-22.7)	**0.052**
above normal, n (%)	4 (12.50)	1 (6.25)	3 (18.75)	0.600
LAVi (mL/m^2^), median (IQR)	41.8 (36.6-52.5)	38.5 (31.5-51.4)	43.0 (37.9-53.0)	0.300
above normal, n (%)	25 (78.13)	11 (68.75)	14 (87.50)	0.394
LV mass				
LVMi (g/m^2^), median (IQR)	96 (85-115)	99 (87-123)	94 (83-111)	0.207
above normal, n (%)	15 (46.88)	7 (43.75)	8 (50.00)	0.999
Diastolic function				
E’med. (cm/s), median (IQR)	6 (5-9)	6 (5-9)	7 (5-9)	0.789
below normal, n (%)	17 (53.13)	9 (56.25)	8 (50.00)	0.999
E’lat * (cm/s), median (IQR)	9 (7-11)	10 (7-11)	8 (7-10)	0.340
below normal, n (%)	14 (51.85)	6 (42.86)	8 (61.54)	0.449
E/E’, median (IQR)	10 (8-12)	8 (7-12)	10 (9-12)	0.392
above normal, n (%)	16 (50.00)	6 (37.50)	10 (62.50)	0.144
E/A, median (IQR)	0.9 (0.7-1.2)	0.9 (0.7-1.0)	0.9 (0.7-1.3)	0.361
abnormal, n (%)	10 (31.25)	5 (31.25)	5 (31.25)	0.999
Systolic function				
EF (%), median (IQR)	64 (61-67)	62 (58-65)	66 (63-68)	**0.005**
abnormal, n (%)	0 (0.00)	0 (0.00)	0 (0.00)	0.999
GLS (%), median (IQR)	-20.0 (-21.4 - -18.4)	-18.5 (-20.9 - -15.9)	-20.2 (-21.5 - - 19.4)	0.062
abnormal, n (%)	16 (50.00)	10 (62.50)	6 (37.50)	0.289

*no data for 5 patients (2 men and 3 women), IQR, interquartile range; p for U – Mann-Whitney test to compare numerical variables between genders, or p for Fisher exact test to compare categorical variables between genders. The bold values are statistically significant.

### Left ventricular myocardial strain assessed by STE

**Figure 3:**
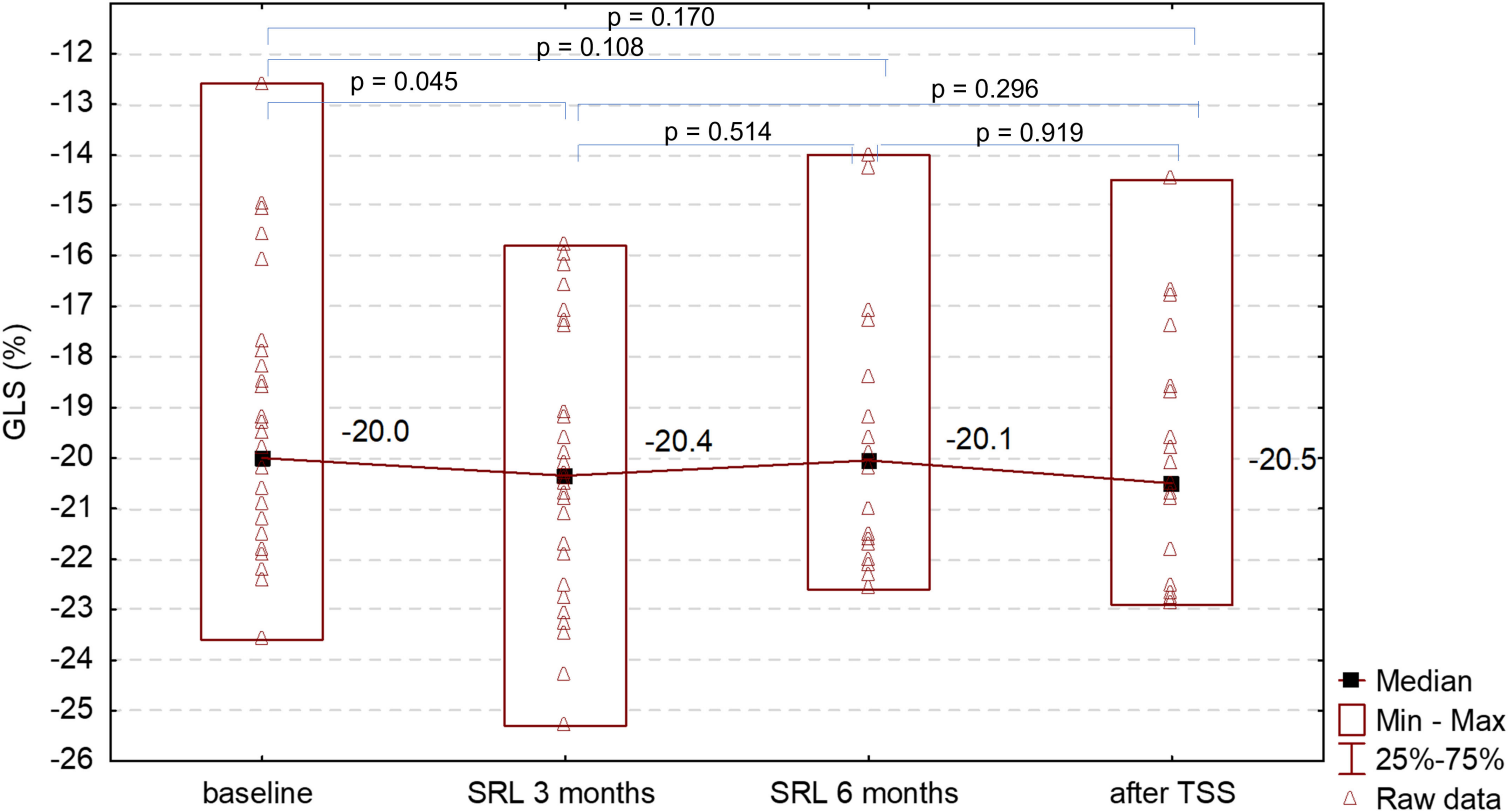
Changes of GLS at baseline, after 3 and 6 months of SRL treatment and 3 months after TSS.

**Figure 4:**
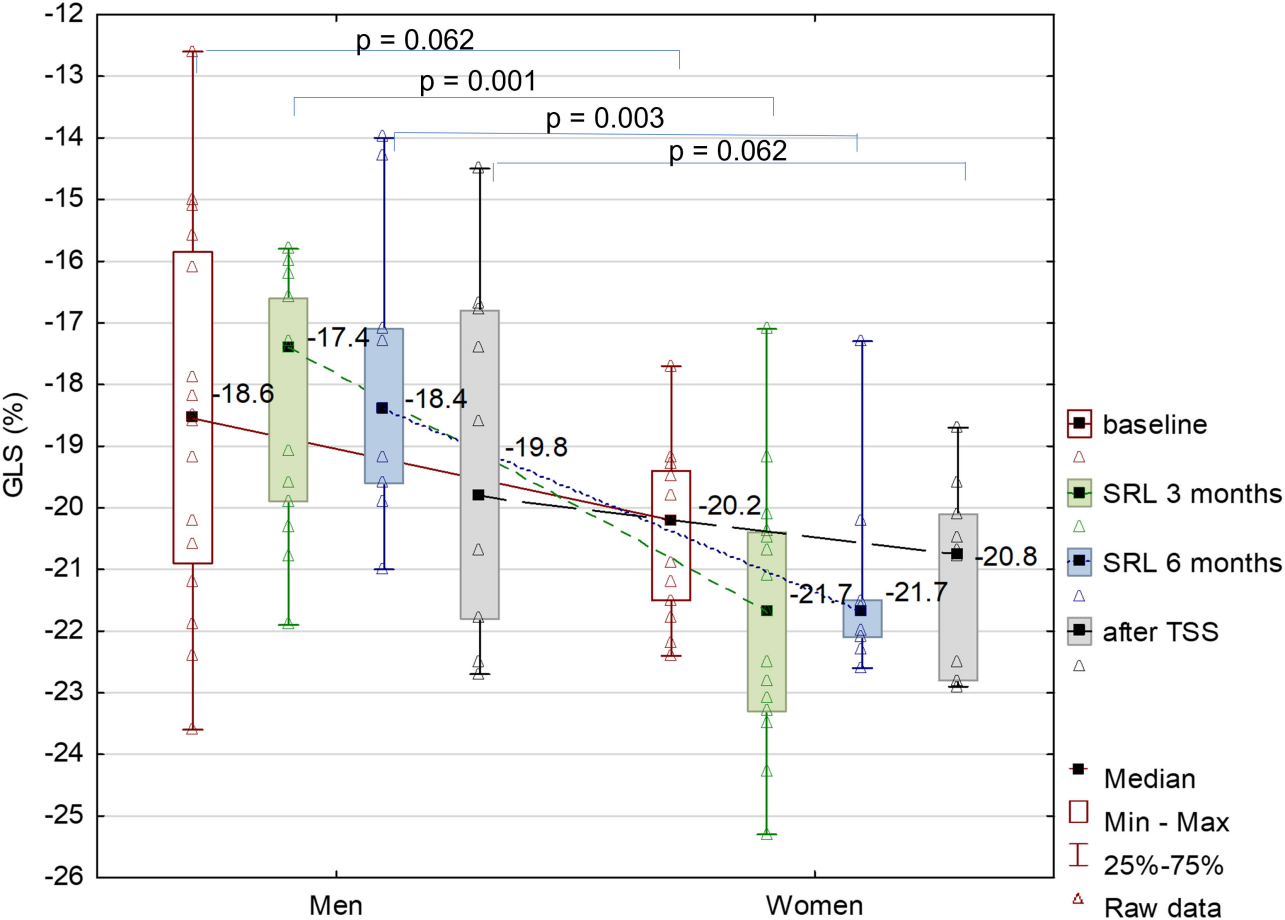
GLS at baseline, after 3 and 6 months of SRL treatment and 3 months after TSS, by gender.

At baseline 50% of newly diagnosed patients with acromegaly had abnormal GLS, i.e., higher than -20%. The majority of patients with abnormal GLS constituted men (62.5% vs. 37.5%). GLS significantly improved after 3 months of SRL treatment comparing to baseline: -20.4% vs. -20.0%, respectively, p=0.045. There was no further change in GLS after 6 months of SRL therapy and no significant change after TSS. The number of patients with abnormal GLS decreased at the end of the study to 43%. The change in GLS during acromegaly treatment is presented in **Figure 3** and in **Supplementary Figure 2**. The median baseline GLS was lower in females compared to males: -20.2% vs. -18.6%, however the difference did not reach the statistical significance (p=0.06). Women had significantly lower GLS than men after 3 and 6 months of SRL treatment (**Figure 4**). There was no statistically significant difference in GLS between women with hypogonadism and women with normal gonadal function either at baseline (p=0.24) or during treatment with SRL (3 and 6 months, respectively: p=0.18, p=0.25) and 3 months after TSS (p=0.92).

### Correlations between GLS and clinical and hormonal characteristics

We found a significant positive correlation of baseline GLS with BMI (r=0.446, p=0.011) and BSA (r=0.411, p=0.019). It means that the higher the patient’s BMI and BSA was, the worse their GLS was, on average. We did not find any statistical correlation of baseline GLS with age, disease duration, arterial hypertension, diabetes status or disease activity at baseline (p>0.05) (**Table 3**).

**Table 3:** Correlations of GLS (%) with characteristics and hormonal parameters in acromegalic patients.

Variable	Test	Baseline		SRL 3 months		SRL 6 months		After TSS	
		test	p	test	p	test	p	test	p
Age (years)	r	0.185	0.311		n/a		n/a		n/a
Sex (males vs females)	U		0.062		**0.001**		**0.003**		0.062
BMI (kg/m^2^)	r	0.446	**0.011**		n/a		n/a		n/a
Disease duration (years)	r	0.210	0.248		n/a		n/a		n/a
BSA (m^2^)	r	0.411	**0.019**		n/a		n/a		n/a
Arterial hypertension (yes vs no)	U		0.281		0.129		0.068		0.944
Diabetes status (diabetes, no, prediabetes)	H		0.612		0.847		0.363		0.796
Dyslipidemia (yes vs no)	U		0.459		0.924		0.815		0.823
Carotid arteries (atherosclerosis vs normal)	U		0.276		0.181		0.796		0.874
Fasting GH (ng/mL)	r	0.051	0.782	-0.035	0.859	0.290	0.294	0.015	0.948
Nadir GH during 75g OGTT (ng/mL)	r	-0.041	0.826		*		*	0.009	0.971
IGF-1 (ng/mL)	r	0.002	0.993	0.056	0.777	0.423	0.117	0.568	**0.007**
IGF-1 (x ULN)	r	0.066	0.721	0.151	0.444	0.446	0.096	0.570	**0.007**

n/a – not applied for time-dependent variables, * - not measured, SRL, somatostatin receptor ligand; TSS, transsphenoidal surgery,

r – Pearson correlation coefficient, U – Mann-Whitney test, H – Kruskal-Wallis test. The bold values are statistically significant.

**Figure 5:**
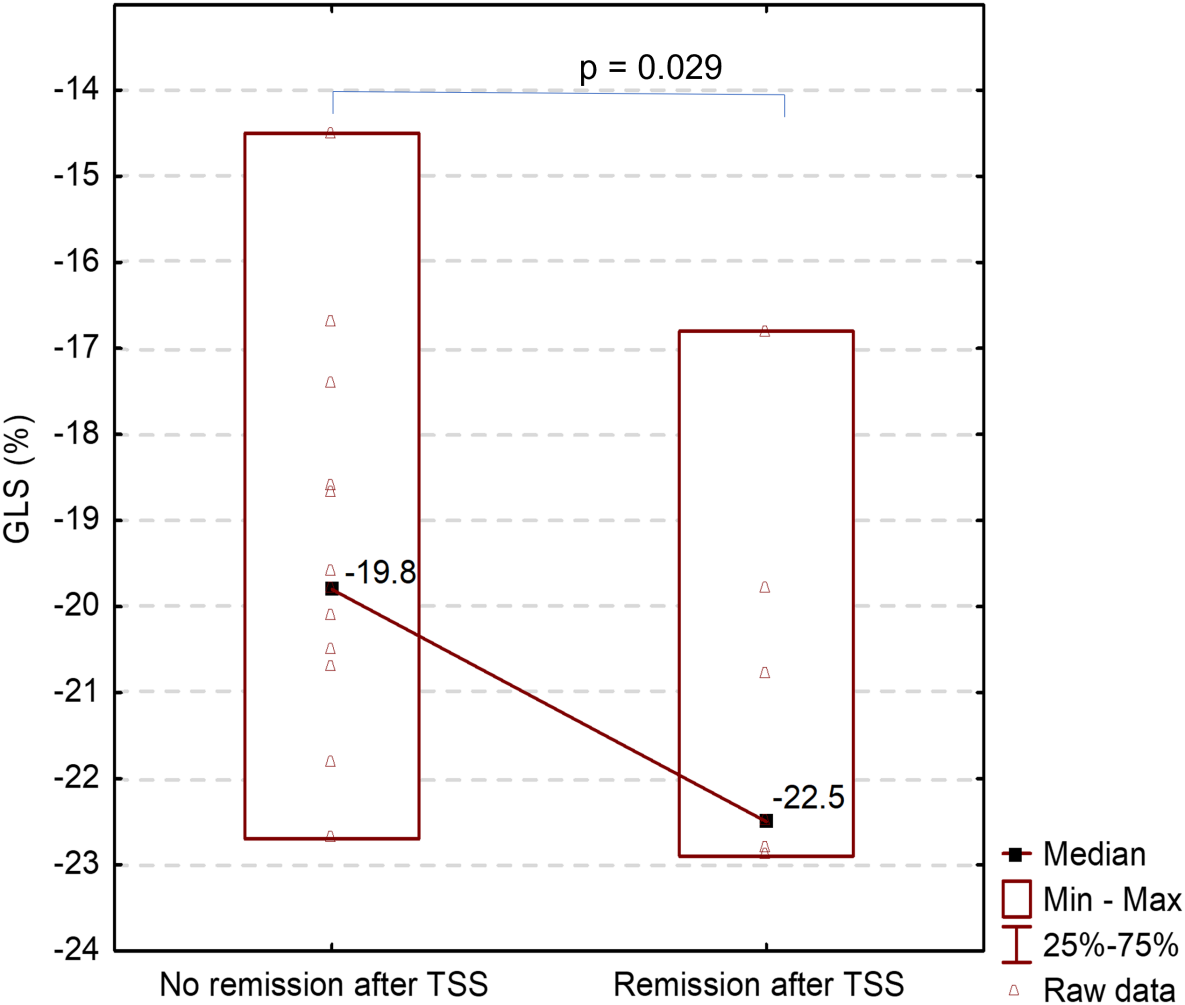
Comparison of GLS after TSS between patients with and without remission after TSS.

There was also a strong positive correlation between GLS and IGF-1 levels after TSS (r=0.570, p=0.007) (**Table 3**). It means that the higher the patient’s IGF-1was, the worse their GLS was, on average. The median GLS was significantly lower in patients who achieved remission after TSS compared to those who did not normalize GH and IGF-1 levels: -22.5% vs -19.8%, respectively, p=0.029 (**Figure 5**).

### Correlations between GLS and baseline echocardiography parameters

**Figure 6:**
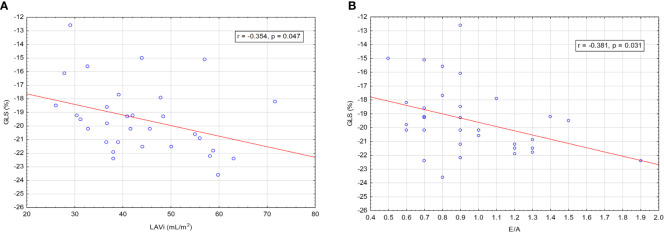
**(A)** Correlation between GLS and LAVi. **(B)** Correlation between GLS and E/A.

We found a significant negative correlation between baseline GLS and LAVi: r= -0.354, p=0.047 (**Figure 6A**) and a negative correlation between GLS and E/A: r= -0.381, p=0.031 (**Figure 6B**). It means that the higher the patient’s LAVi and E/A were, the more negative their GLS was, which means better, on average. We did not find any significant correlations between GLS and other echocardiographic parameters, including LVMi, at baseline (**Table 4**).

**Table 4:** Correlations of GLS (%) with echocardiography parameters in acromegalic patients, at baseline.

Variable	r	p
Cardiac chamber size		
LVEDV (mL)	0.231	0.204
LVEDV/BSA (mL/m^2^)	0.054	0.768
LVESV (mL)	0.305	0.089
LVESV/BSA (mL/m^2^)	0.163	0.373
LAVi (mL/m^2^)	-0.354	**0.047**
LV mass		
LVMi (g/m^2^)	0.192	0.292
Diastolic function		
E’med. (cm/s)	-0.265	0.142
E’lat (cm/s)	0.138	0.493
E/E’	-0.010	0.978
E/A	-0.381	**0.031**
Systolic function		
EF (%)	-0.538	**0.001**

r – Pearson correlation coefficient. The bold values are statistically significant.

## Discussion

Our study confirmed the beneficial impact of acromegaly treatment on LV systolic function assessed by STE. The median baseline GLS in the study group was normal and amounted to -20%. However, it is worth emphasizing that 50% of the subjects had an abnormal value of this parameter which indicated subclinical LV systolic dysfunction. In our recent study, retrospectively evaluating a cohort of 43 previously untreated acromegaly patients with normal EF, the median GLS value was significantly worse compared to the control group (-16.6% vs. -20.7%; p < 0.01) and that was consistent with subclinical systolic dysfunction (21). The better GLS values observed in the current prospective study could be probably attributed to the fact that the mean LVMi was normal in both male and female groups: 99g/m^2^ and 94g/m^2^, respectively, though almost 47% of patients had LVH. In the studies available in the literature, where abnormal GLS in acromegaly patients was found, the LVMi values were definitely higher than in our group of patients (18, 21).

Another reasons that could explain the normal median GLS result and normal median LVMi were possibly the good hypertension control and the cardioprotective effect of novel antihypertensive drugs which has been proven in many studies (27–29). In our study, in the group of 17 patients diagnosed with hypertension, 14 (82.3%) were treated with ACEI/ARB-the drugs that have the potential to reverse LVH. Interestingly, in our study women had significantly better GLS values not only baseline but also during acromegaly treatment compared to men. This fact may be partly explained by the lower BMI and BSA values in women compared to men: 26 kg/m^2^ vs. 29 kg/m^2^, p<0.001 and 1.8 m^2^ vs. 2.2 m^2^, p<0.001, respectively and a strong positive correlation between these parameters and GLS.

Women with acromegaly, like women in the general population, have better GLS compared to men (30). Similar observations had researchers evaluating the heart of athletes by STE. Female athletes have also been found to have better GLS than male athletes suggesting that further research is needed in STE and gender differences (31).

These results are surprising especially when we realize that women with acromegaly, but also with other cardiovascular diseases have a worse prognosis and shorter life expectancy (7). A Korean nationwide study based on 718 acromegaly patients confirmed a significantly increased mortality risk in females (HR=1.75) but not in males (4). It is not fully known whether gender affects acromegaly, but it has been proven that some diseases show sexual dimorphism. The gonadal steroids modulate the GH axis, androgens enhance the effects of GH, and in contrast, estrogens exert an opposite effect by inhibiting the production of IGF-1 from the liver (32–34). Lenders et al. wrote about a different biochemical profile in women and men with acromegaly (35). This profile in women consists of relatively higher GH levels, which causes insulin resistance, and lower IGF-1 levels, which mediates anabolism. These differences can cause higher prevalence of hypertension and diabetes in women, and what is very important, they can be the cause of longer delay in diagnosis in women (35, 36). The lower IGF-1 levels in females with acromegaly may be also the reason for better GLS values. In our study females had significantly lower IGF-1 levels compared to males not only baseline but also during treatment.

In the light of the previous data on healthy subjects and our results, it may be necessary to redefine and differentiate the cut-off points for women and men for the GLS parameter, similarly to the different normal ranges used for other echocardiographic parameters.

Pierre et al. wrote that it was necessary, because a woman’s heart differed not only in size but above all, it had a different microstructural architecture (37).

The female heart has a larger EF and beats at a faster rate but generates a smaller cardiac output. It has a lower blood pressure but produces universally larger contractile strains (37).

The sex differences in cardiac form and function are complex, important, and obvious, and can’t be ignored. When we use similar diagnostic criteria for female and male hearts, cardiac diseases in women can be overlooked, diagnosed later and with more severe symptoms than in men. This should be applied also to females with acromegaly to improve their increased cardiovascular mortality compared to males.

To the best of our knowledge this is the first prospective study to evaluate the effect of acromegaly treatment on GLS value, not only the effect of SRL treatment but also the effect of TSS. Silva et al. and Bogazzi et al. in prospective studies evaluated patients with acromegaly before and after 12 months of octreotide and 6 months of lanreotide treatment (38, 39). However, the results of SRL treatment on the heart were conflicting.

In the above-mentioned studies, echocardiography and cardiac magnetic resonance imaging (CMRI) were used to assess changes in the patients’ heart. Silva et al. in a group of 40 patients (30 were reevaluated after 12 months) did not find any clinically relevant differences in cardiac variables after 12 months of acromegaly treatment. Interestingly, only 5% of patients had LV hypertrophy features in CMRI versus 31% in echocardiography (38). In turn, Bogazzi et al. in a smaller group of 14 acromegaly patients, found a significant reduction in LVMi in the CMRI after 6 months of SRL treatment, which was even more evident when the disease was biochemically controlled (39). Colao et al. assessed the early effect of octreotide LAR treatment on echocardiography parameters in 15 patients and among them 10 patients had previously undergone TSS. In this study more serious abnormalities in the heart of patients were observed baseline: advanced LVH and systolic dysfunction assessed by reduced EF. In the Colao et al. study a significant reduction in LVMi was obtained in all patients, with a resolution of LVH in 6 out of 11 patients. In 5 acromegalics (33%) who had reduced EF at baseline, normalization of EF was achieved in the course of treatment. Additionally, in the patients who achieved disease control - in 9 out of 15, an improvement of exercise duration and capacity was observed (40).

Hypersecretion of GH appears to have a direct effect on the heart and is associated with a specific cardiomyopathy that results in structural and functional abnormalities due to interstitial fibrosis and edema. It is well known that GH affects water balance, and that GH excess increases myocardial water content. Gouya et al. showed that increased myocardial T2 relaxation time in CMRI which indicates myocardial edema can be normalized soon after effective treatment (10 days for seven patients) and is significantly correlated with successful reduction of GH and IGF-1 levels (41). We showed in our study that after 3 months of SRL treatment there was a significant improvement in GLS values. During this period, there was also the greatest reduction in IGF-1 levels. Therefore, the initiation of medical treatment should not be delayed, especially if the patient must wait for TSS. We also showed that complete transsphenoidal adenomectomy should always be aimed at - in this group of patients, GLS values were significantly lower than in non-radically operated patients. We also noticed a significant correlation between IGF-1 levels and GLS values.

The beneficial effect of acromegaly treatment could explain the discrepancies in GLS in recent studies, where STE was performed in patients who previously underwent TSS and were treated with SRL. Volschan et al. and Gadelha et al. did not find a significant differences in GLS between acromegaly patients and control groups. However, 62.2% of the patients enrolled to the Volschan et al. study were during acromegaly treatment, and 80% of the patients in Gadelha et al. study had a history of TSS and 72% were using medical therapy (19, 20). In recent studies a very similar picture of the “acromegalic heart” is presented with normal EF, varying degrees of LVH, usually mildly expressed diastolic dysfunction, and significantly enlarged LA (8, 18, 19, 21).

Almost 80% of our patients had an enlarged LA with mild diastolic dysfunction (in about 50% of patients) and with LVH occurring in less than 50% of patients, however the mean LVMi was normal in both male and female groups. It is difficult to explain such changes only by an increase in the afterload. Some similarities can be found with an athlete’s heart in which atrial remodeling is a physiological adaptation to volume overload allowing for greater volume delivery and increased cardiac output (42).

In patients with acromegaly, volume overload may result from the action of GH leading to water and sodium retention (36). When we look for similarities between the “acromegalic heart” and the athlete’s heart, it is important to remember that hypertrophy of the LV in athletes occurs just through the activation of the IGF-1 pathway, unlike in pathological hypertrophy where the angiotensin 1 pathway is activated (43).

The LA has been incorrectly perceived as a simple transport chamber for many years.

Currently, its hemodynamic function is known in three integrated phases:1. reservoir 2. conduit and 3. booster-pump. Similarly to the LV, the Frank-Starling mechanism works in the LA. The increase in blood volume stretches the myocardial fibers, causing the cardiac muscle to contract more forcefully with increase in its mechanical performance (44).

In our study, we showed a negative correlation between LAVi and GLS. It may indicate that the dynamic and interactive relationship between LA and LV in the heart of an acromegaly patient is similar to that of an athlete’s heart. Nevertheless, atrial fiber shortening, and contractility begin to fail with progressive dilation of LA. It means the threshold fiber length has been reached and further enlargement will only result in a decline of atrial function (44). Regardless of the mechanisms by which LA enlargement occurs, it results in an increased risk of arrhythmias, including atrial fibrillation, stroke, mitral regurgitation and heart failure.

Therefore, people with the so-called healthy heart and enlarged LA, like athletes, should be periodically examined using the STE method to evaluate both the LV and the LA (45) and they should have Holter ECG periodically performed (46). It is known from many studies that abnormal GLS in patients with preserved EF is associated with an increased risk of cardiac hospitalization and cardiovascular mortality (12). However, it is still an open question whether abnormal GLS in acromegalic patients with normal EF changes the prognosis of these patients, assuming that they are properly treated for the underlying disease and comorbidities. We are aware that a short follow up duration is a limitation of our study.

We expect all our patients to improve or at least maintain their results in the further follow up, and we are particularly interested in the group of patients with abnormal GLS values, whether they will maintain a normal EF after 5 years of follow-up.

## Conclusions

To our knowledge, this is the first prospective study using GLS as a marker to assess the impact of a comprehensive medical and surgical treatment on LV function in patients with acromegaly, taking into account sex differences. Half of the newly diagnosed patients with acromegaly presented subclinical LV systolic dysfunction assessed by STE. However, the beneficial effect of acromegaly treatment on the LV systolic function was noticeable already after 3 months of SRL therapy, especially in females who had better GLS values compared to males during medical treatment. Surgical remission should always be aimed at, because surgical remission leads to better systolic function assessed by STE in acromegaly patients.

## Data availability statement

The raw data supporting the conclusions of this article will be made available by the authors, without undue reservation.

## Ethics statement

The studies involving human participants were reviewed and approved by Bioethics Committee at The Centre of Postgraduate Medical Education, Warsaw, Poland. The patients/participants provided their written informed consent to participate in this study.

## Author contributions

AP-G and MS-B designed the study, enrolled the patients, analyzed the data, and wrote the manuscript. MS-B and IC-O were involved in managing the patients. AP-G performed all 2D-Echocardiography and STE studies. GZ performed pituitary surgeries. IC-O, WZ, MD, and WK critically revised the manuscript. DR performed the statistical analysis. All authors approved the final version. All authors contributed to the article.

## Supplementary material

Supplementary Figure 1
**(A).** Individual changes of GH at baseline, after 3 and 6 months of SRL treatment, 3 months after TSS. **(B).** Individual changes of IGF-1 at baseline, after 3 and 6 months of SRL treatment, 3 months after TSS. **(C).** Individual changes of IGF-1xULN at baseline, after 3 and 6 months of SRL treatment and 3 months after TSS.

Supplementary Figure 2 Individual changes of GLS at baseline, after 3 and 6 months of SRL treatment and 3 months after TSS.

## References

[1] Giustina A Barkan A Beckers A Biermasz N Biller BMK Boguszewski C. A consensus on the diagnosis and treatment of acromegaly comorbidities: an update. J Clin Endocrinol Metab (2020) 105(4):dgz096. doi: 10.1210/clinem/dgz096 31606735

[2] Ritvonen E Löyttyniemi E Jaatinen P Ebeling T Moilanen L Nuutila P. Mortality in acromegaly: a 20-year follow-up study. Endocr Relat Cancer (2016) 23(6):469–80. doi: 10.1530/ERC-16-0106 27185871

[3] Maione L Brue T Beckers A Delemer B Petrossians P Borson-Chazot F. Changes in the management and comorbidities of acromegaly over three decades: the French acromegaly registry. Eur J Endocrinol (2017) 176(5):645–55. doi: 10.1530/EJE-16-1064 28246150

[4] Park KH Lee EJ Seo GH Ku CR. Risk for acromegaly-related comorbidities by sex in Korean acromegaly. J Clin Endocrinol Metab (2020) 105(4):dgz317. doi: 10.1210/clinem/dgz317 31903478

[5] Gadelha MR Kasuki L Lim DST Fleseriu M. Systemic complications of acromegaly and the impact of the current treatment landscape: an update. Endocr Rev (2019) 40(1):268–332. doi: 10.1210/er.2018-00115 30184064

[6] Bihan H Espinosa C Valdes-Socin H Salenave S Young J Levasseur S. Long-term outcome of patients with acromegaly and congestive heart failure. J Clin Endocrinol Metab (2004) 89(11):5308–13. doi: 10.1210/jc.2004-0821 15531475

[7] Mosca S Paolillo S Colao A Bossone E Cittadini A Iudice FL. Cardiovascular involvement in patients affected by acromegaly: an appraisal. Int J Cardiol (2013) 167(5):1712–8. doi: 10.1016/j.ijcard.2012.11.109 23219077

[8] Popielarz-Grygalewicz A Gąsior JS Konwicka A Grygalewicz P Stelmachowska-Banaś M Zgliczyński W. Heart in acromegaly: the echocardiographic characteristics of patients diagnosed with acromegaly in various stages of the disease. Int J Endocrinol (2018) 2018:6935054. doi: 10.1155/2018/6935054 30123265 PMC6079421

[9] Tops LF Delgado V Marsan NA Bax JJ. Myocardial strain to detect subtle left ventricular systolic dysfunction. Eur J Heart Fail (2017) 19(3):307–13. doi: 10.1002/ejhf.694 27891719

[10] Stampehl MR Mann DL Nguyen JS Cota F Colmenares C Dokainish H. Speckle strain echocardiography predicts outcome in patients with heart failure with both depressed and preserved left ventricular ejection fraction. Echocardiography (2015) 32(1):71–8. doi: 10.1111/echo.12613 24816065

[11] Smiseth OA Torp H Opdahl A Haugaa KH Urheim S. Myocardial strain imaging: how useful is it in clinical decision making? Eur Heart J (2016) 37(15):1196–207. doi: 10.1093/eurheartj/ehv529 PMC4830908 26508168

[12] Verdonschot JAJ Henkens M Wang P Schummers G Raafs AG Krapels IPC. A global longitudinal strain cut-off value to predict adverse outcomes in individuals with a normal ejection fraction. ESC Heart Fail (2021) 8(5):4343–5. doi: 10.1002/ehf2.13465 PMC8497344 34272829

[13] Kalam K Otahal P Marwick TH. Prognostic implications of global LV dysfunction: a systematic review and meta-analysis of global longitudinal strain and ejection fraction. Heart (2014) 100(21):1673–80. doi: 10.1136/heartjnl-2014-305538 24860005

[14] Thavendiranathan P Poulin F Lim KD Plana JC Woo A Marwick TH. Use of myocardial strain imaging by echocardiography for the early detection of cardiotoxicity in patients during and after cancer chemotherapy: a systematic review. J Am Coll Cardiol (2014) 63(25 Pt A):2751–68. doi: 10.1016/j.jacc.2014.01.073 24703918

[15] Uziębło-Życzkowska B Krzesinński P Witek P Zielinński G Jurek A Gielerak G. Cushing's disease: subclinical left ventricular systolic and diastolic dysfunction revealed by speckle tracking echocardiography and tissue Doppler imaging. Front Endocrinol (Lausanne) (2017) 8:222. doi: 10.3389/fendo.2017.00222 28928716 PMC5591890

[16] Mihaila S Mincu RI Rimbas RC Dulgheru RE Dobrescu R Magda SL. Growth hormone deficiency in adults impacts left ventricular mechanics: a two-dimensional speckle-tracking study. Can J Cardiol (2015) 31(6):752–9. doi: 10.1016/j.cjca.2015.01.008 26022988

[17] Yang QM Fang JX Chen XY Lv H Kang CS. The systolic and diastolic cardiac function of patients with type 2 diabetes mellitus: an evaluation of left ventricular strain and torsion using conventional and speckle tracking echocardiography. Front Physiol (2021) 12:726719. doi: 10.3389/fphys.2021.726719 35069231 PMC8777120

[18] Uzibło-Życzkowska B Jurek A Witek P Zieliński G Gielerak G Krzesiński P. Left heart dysfunction in acromegaly revealed by novel echocardiographic methods. Front Endocrinol (Lausanne) (2020) 11:418. doi: 10.3389/fendo.2020.00418 32670201 PMC7326767

[19] Gadelha P Santos ECL Castillo J Vilar L. Subclinical ventricular dysfunction in long-term acromegaly assessed by speckle-tracking echocardiography. Front Endocrinol (Lausanne) (2022) 13:812964. doi: 10.3389/fendo.2022.812964 35185796 PMC8854639

[20] Volschan ICM Kasuki L Silva CMS Alcantara ML Saraiva RM Xavier SS. Two-dimensional speckle tracking echocardiography demonstrates no effect of active acromegaly on left ventricular strain. Pituitary (2017) 20(3):349–57. doi: 10.1007/s11102-017-0795-9 28220351

[21] Popielarz-Grygalewicz A Stelmachowska-Banaś M Gąsior JS Grygalewicz P Czubalska M Zgliczyński W. Subclinical left ventricular systolic dysfunction in patients with naive acromegaly - assessment with two-dimensional speckle-tracking echocardiography: retrospective study. Endokrynol Pol (2020) 71(3):227–34. doi: 10.5603/EP.a2020.0021 32293699

[22] American Diabetes Association Professional Practice Committee. 2. classification and diagnosis of diabetes: standards of medical care in diabetes-2022. Diabetes Care (2022) 45(Suppl 1):S17–38. doi: 10.2337/dc22-S002 34964875

[23] Lang RM Badano LP Mor-Avi V Afilalo J Armstrong A Ernande L. Recommendations for cardiac chamber quantification by echocardiography in adults: an update from the American society of echocardiography and the European association of cardiovascular imaging. J Am Soc Echocardiogr (2015) 28(1):1–39.e14. doi: 10.1016/j.echo.2014.10.003 25559473

[24] Nagueh SF Smiseth OA Appleton CP Byrd BF 3rd Dokainish H Edvardsen T. Recommendations for the evaluation of left ventricular diastolic function by echocardiography: an update from the American society of echocardiography and the European association of cardiovascular imaging. J Am Soc Echocardiogr (2016) 29(4):277–314. doi: 10.1016/j.echo.2016.01.011 27037982

[25] Johri AM Nambi V Naqvi TZ Feinstein SB Kim ESH Park MM. Recommendations for the assessment of carotid arterial plaque by ultrasound for the characterization of atherosclerosis and evaluation of cardiovascular risk: from the American society of echocardiography. J Am Soc Echocardiogr (2020) 33(8):917–33. doi: 10.1016/j.echo.2020.04.021 32600741

[26] Elwertowski M Małek G. Standards of the polish ultrasound society - update. examination of extracranial carotid and vertebral arteries. J Ultrason (2014) 14(57):179–91. doi: 10.15557/JoU.2014.0018 PMC4579696 26673158

[27] Mizuguchi Y Oishi Y Miyoshi H Iuchi A Nagase N Oki T. Beneficial effects of telmisartan on left ventricular structure and function in patients with hypertension determined by two-dimensional strain imaging. J Hypertens (2009) 27(9):1892–9. doi: 10.1097/HJH.0b013e32832d8785 19506525

[28] Thomas JDJ Dattani A Zemrak F Burchell T Akker SA Kaplan FJL. Renin-angiotensin system blockade improves cardiac indices in acromegaly patients. Exp Clin Endocrinol Diabetes (2017) 125(6):365–7. doi: 10.1055/s-0042-123710 PMC6193280 28166592

[29] Chen JS Pei Y Li CE Li YN Wang QY Yu J. Comparative efficacy of different types of antihypertensive drugs in reversing left ventricular hypertrophy as determined with echocardiography in hypertensive patients: a network meta-analysis of randomized controlled trials. J Clin Hypertens (Greenwich) (2020) 22(12):2175–83. doi: 10.1111/jch.14047 PMC8029902 33190366

[30] Li G Zhang Z Gao Y Zhu C Zhou S Cao L. Age- and sex-specific reference values of biventricular strain and strain rate derived from a large cohort of healthy Chinese adults: a cardiovascular magnetic resonance feature tracking study. J Cardiovasc Magn Reson (2022) 24(1):63. doi: 10.1186/s12968-022-00881-1 36404299 PMC9677678

[31] Forsythe L George K Oxborough D. Speckle tracking echocardiography for the assessment of the athlete's heart: is it ready for daily practice? Curr Treat Options Cardiovasc Med (2018) 20(10):83. doi: 10.1007/s11936-018-0677-0 30146663 PMC6132779

[32] Meinhardt UJ Ho KK. Modulation of growth hormone action by sex steroids. Clin Endocrinol (Oxf) (2006) 65(4):413–22. doi: 10.1111/j.1365-2265.2006.02676.x 16984231

[33] Weissberger AJ Ho KK. Activation of the somatotropic axis by testosterone in adult males: evidence for the role of aromatization. J Clin Endocrinol Metab (1993) 76(6):1407–12. doi: 10.1210/jcem.76.6.8501143 8501143

[34] Ho KY Evans WS Blizzard RM Veldhuis JD Merriam GR Samojlik E. Effects of sex and age on the 24-hour profile of growth hormone secretion in man: importance of endogenous estradiol concentrations. J Clin Endocrinol Metab (1987) 64(1):51–8. doi: 10.1210/jcem-64-1-51 3782436

[35] Lenders NF McCormack AI Ho KKY. MANAGEMENT OF ENDOCRINE DISEASE: does gender matter in the management of acromegaly? Eur J Endocrinol (2020) 182(5):R67–r82. doi: 10.1530/EJE-19-1023 32069216

[36] Yang H Tan H Huang H Li J. Advances in research on the cardiovascular complications of acromegaly. Front Oncol (2021) 11:640999. doi: 10.3389/fonc.2021.640999 33869029 PMC8050332

[37] St Pierre SR Peirlinck M Kuhl E. Sex matters: a comprehensive comparison of female and Male hearts. Front Physiol (2022) 13:831179. doi: 10.3389/fphys.2022.831179 35392369 PMC8980481

[38] dos Santos Silva CM Gottlieb I Volschan I Kasuki L Warszawski L Balarini Lima GA. Low frequency of cardiomyopathy using cardiac magnetic resonance imaging in an acromegaly contemporary cohort. J Clin Endocrinol Metab (2015) 100(12):4447–55. doi: 10.1210/jc.2015-2675 26431508

[39] Bogazzi F Lombardi M Strata E Aquaro G Lombardi M Urbani C. Effects of somatostatin analogues on acromegalic cardiomyopathy: results from a prospective study using cardiac magnetic resonance. J Endocrinol Invest (2010) 33(2):103–8. doi: 10.1007/BF03346562 20348836

[40] Colao A Marzullo P Ferone D Spinelli L Cuocolo A Bonaduce D. Cardiovascular effects of depot long-acting somatostatin analog sandostatin LAR in acromegaly. J Clin Endocrinol Metab (2000) 85(9):3132–40. doi: 10.1210/jcem.85.9.6782 10999798

[41] Gouya H Vignaux O Le Roux P Chanson P Bertherat J Bertagna X. Rapidly reversible myocardial edema in patients with acromegaly: assessment with ultrafast T2 mapping in a single-breath-hold MRI sequence. AJR Am J Roentgenol (2008) 190(6):1576–82. doi: 10.2214/AJR.07.2031 18492909

[42] Lasocka Z Lewicka-Potocka Z Faran A Daniłowicz-Szymanowicz L Nowak R Kaufmann D. Exercise-induced atrial remodeling in female amateur marathon runners assessed by three-dimensional and speckle tracking echocardiography. Front Physiol (2022) 13:863217. doi: 10.3389/fphys.2022.863217 35860663 PMC9289460

[43] Carbone A D'Andrea A Riegler L Scarafile R Pezzullo E Martone F. Cardiac damage in athlete's heart: when the "supernormal" heart fails! World J Cardiol (2017) 9(6):470–80. doi: 10.4330/wjc.v9.i6.470 PMC5491465 28706583

[44] Mehrzad R Rajab M Spodick DH. The three integrated phases of left atrial macrophysiology and their interactions. Int J Mol Sci (2014) 15(9):15146–60. doi: 10.3390/ijms150915146 PMC4200839 25167138

[45] Cheema B Kinno M Gu D Ryan J Mitter S Rigolin V. Left atrial size and strain in elite athletes: a cross-sectional study at the NBA draft combine. Echocardiography (2020) 37(7):1030–6. doi: 10.1111/echo.14680 32634261

[46] Hong S Kim KS Han K Park CY. Acromegaly and cardiovascular outcomes: a cohort study. Eur Heart J (2022) 43(15):1491–9. doi: 10.1093/eurheartj/ehab822 34864952

