# Supplementary figures and images for: Effects of acromegaly treatment on left ventricular systolic function assessed by speckle tracking echocardiography in relation to sex differences: results from a prospective single center study
Source: Front Endocrinol (Lausanne). 2023 May 8;14:1154615. doi: 10.3389/fendo.2023.1154615 (PMC10200955; doi:10.3389/fendo.2023.1154615)

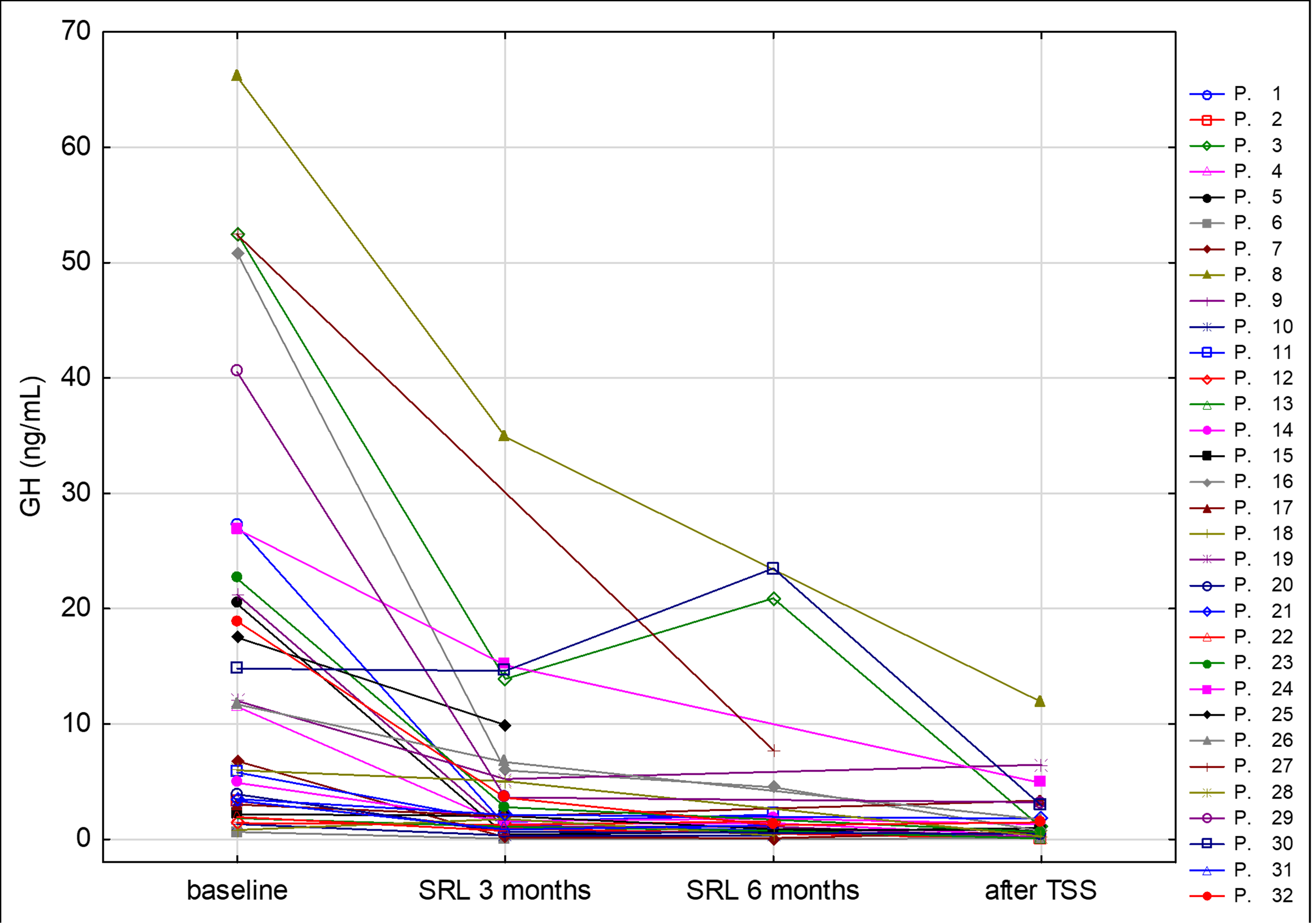

Supplement: Supplementary Figure 1 — (A). Individual changes of GH at baseline, after 3 and 6 months of SRL treatment, 3 months after TSS. (B). Individual changes of IGF-1 at baseline, after 3 and 6 months of SRL treatment, 3 months after TSS. (C). Individual changes of IGF-1xULN at baseline, after 3 and 6 months of SRL treatment and 3 months after TSS. [file Image_1.tif]

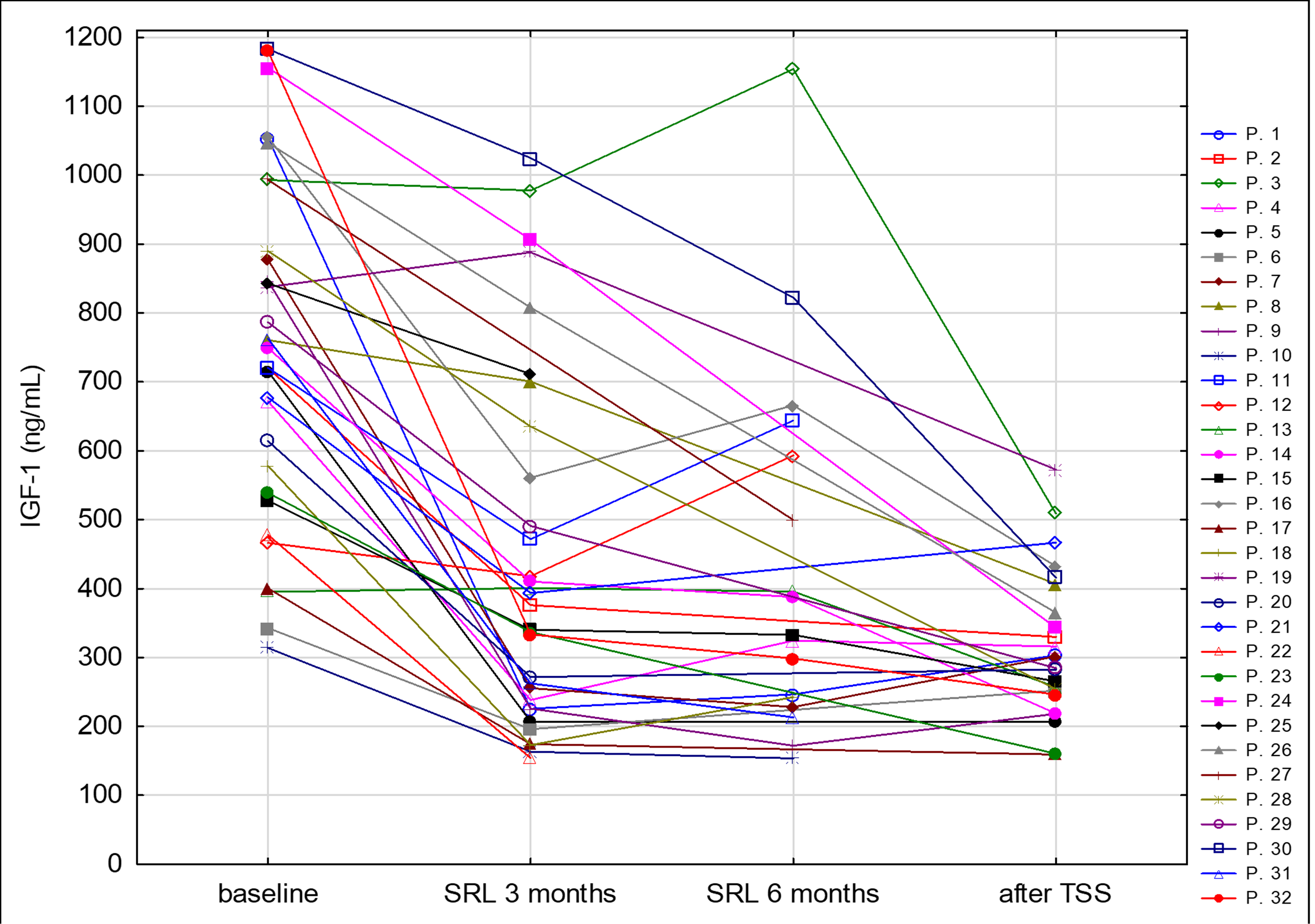

Supplement: Supplementary Figure 2 — Individual changes of GLS at baseline, after 3 and 6 months of SRL treatment and 3 months after TSS. [file Image_2.tif]

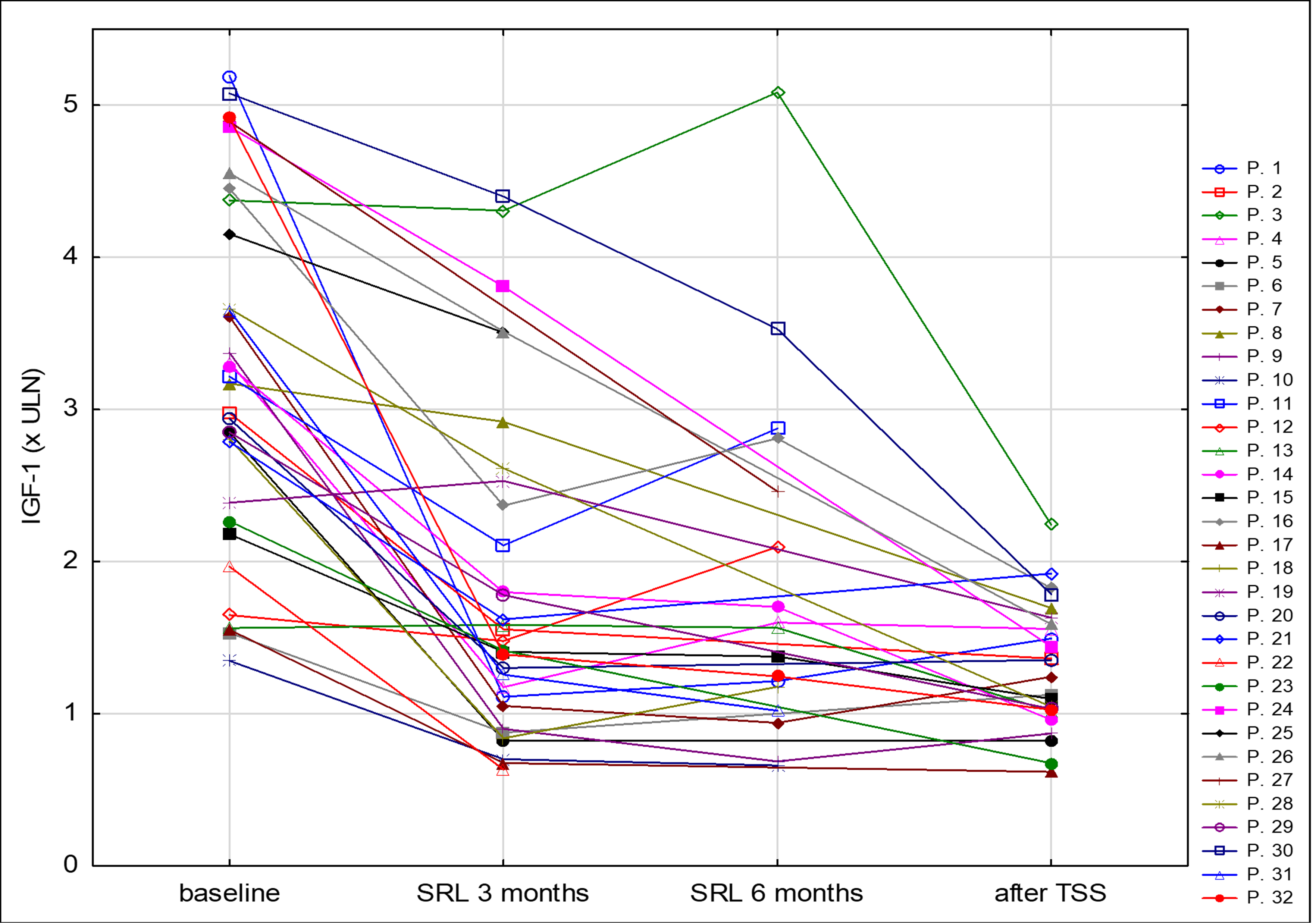

Supplement: Supplementary file 3 [file Image_3.tif]

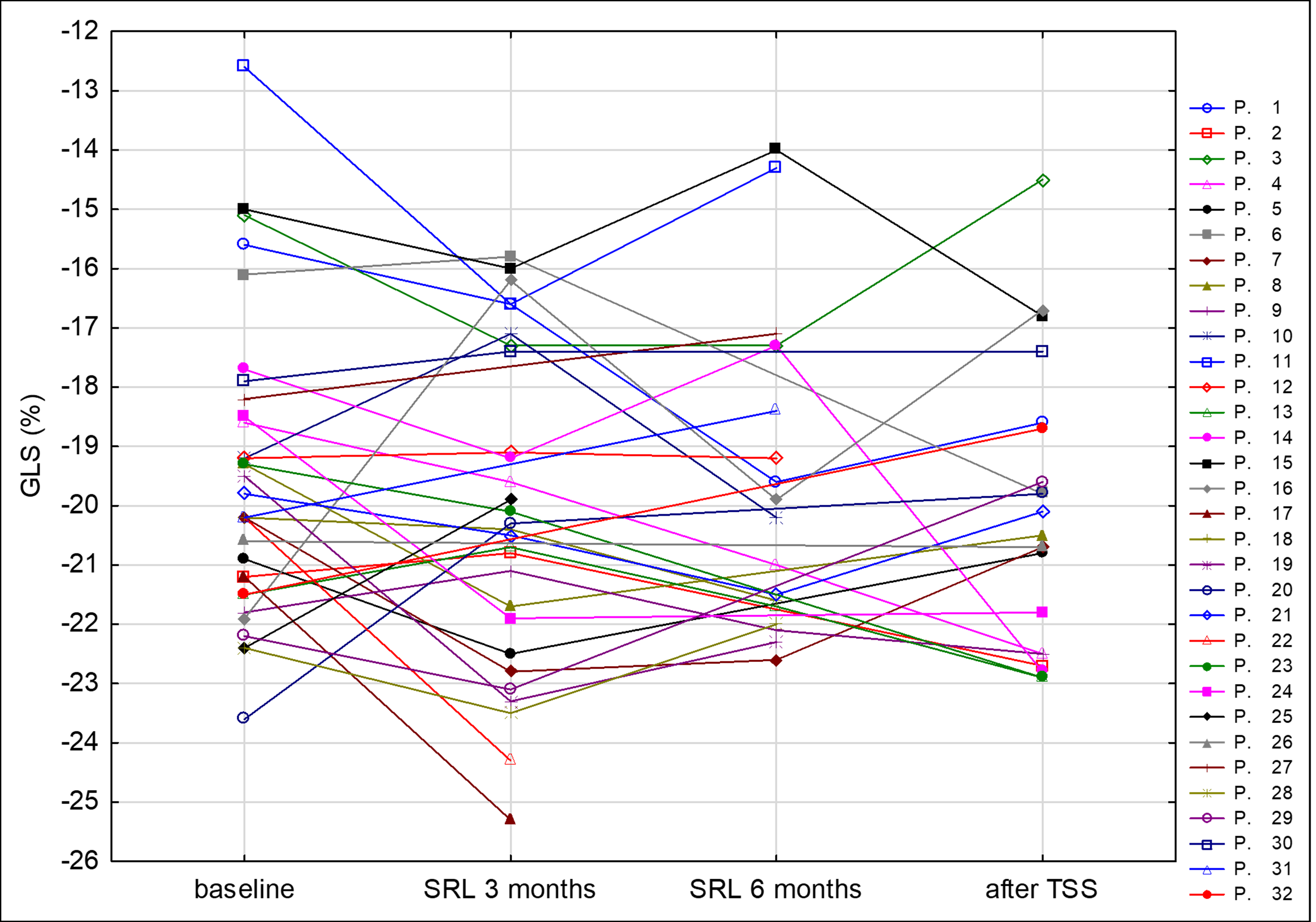

Supplement: Supplementary file 4 [file Image_4.tif]
